# Xanthogranulomatous Oophoritis: A Rare Case Report

**DOI:** 10.7759/cureus.43724

**Published:** 2023-08-18

**Authors:** Pratibha Dawande, Rashmi Wankhade, Milind Pande

**Affiliations:** 1 Pathology, Datta Meghe Medical College, Datta Meghe Institute of Higher Education and Research, Wardha, Nagpur, IND

**Keywords:** macrophages, inflammation, oophoritis, histiocytes, xanthogranulomatous

## Abstract

Xanthogranulomatous oophoritis is a rare, chronic and non-neoplastic condition in which a heavy foamy histiocyte inflammatory infiltrate admixed with neutrophils, plasma cells, multinucleated giant cells, fibroblasts and foci of necrosis causing extensive tissue damage and organ destruction. The gallbladder and kidney are just two examples of the different organs that exhibit histological changes resembling xanthogranulomatous alteration. The present case involved a 40-year-old female who presented with a tuboovarian mass and was ultimately diagnosed with xanthogranulomatous oophritis, despite initial clinicoradiological suspicions for malignancy. Xanthogranulomatous oophritis is a significant entity because, clinically and radiographically, it resembles tumours of the ovary and hinges on a careful histopathological analysis to establish a diagnosis.

## Introduction

A particular type of chronic inflammation known as xanthogranulomatous inflammation causes tissue damage by inflammatory cell infiltrate composed of lymphocytes, plasma cells, macrophages, multinucleated giant cells and neutrophils [1].

Xanthogranulomatous oophoritis has been previously reported in the literature under various names, including xanthogranulomatous salpingitis-oophoritis, xanthogranulomatous inflammation of the ovary, and lipid cell granuloma of the ovary. In 1968 Roth published the first article describing a case of xanthogranulomatous oophoritis and since then, there have been almost 100 cases documented in the literature. Xanthogranulomatous oophoritis has an uncertain specific aetiology, although it's been postulated to result from chronic inflammation, infection, or autoimmune disorders [2].

A unique type of chronic inflammation called xanthogranulomatous inflammation damages the affected tissue. This is an uncommon process that frequently affects the gallbladder and kidney. The stomach, anorectal area, testis, epididymis, urinary bladder, ovaries, fallopian tubes, endometrium and bones have all been implicated in the xanthogranulomatous inflammatory process [3].

Xanthogranulomatous inflammation involving the female genital tract is rare and only impacts the endometrium. In the female genital tract, endometrium, fallopian tubes, or ovaries may be affected locally or completely by xanthogranulomatous inflammation, which, because of its rarity, could be misinterpreted for a malignant lesion and clinically manifests as a tumour in the pelvic cavity that extends to the surrounding tissues [4].

In this case report, we present a rare instance of xanthogranulomatous oophoritis in a 40-year-old female and discuss its clinical presentation and histopathological findings.

## Case presentation

A 40-year-old female presented with the chief complaints of heavy, irregular cycles lasting six months, as well as intermittent abdominal pain. A solid right adnexal mass could be felt during vaginal examination. Her blood tests revealed hemoglobin of 9.4 g/dl, a modest increase in total leukocyte count (14,000/cu mm), a moderate increase in erythrocyte sedimentation rate (44 mm at first hour), and a marginal increase in lactate dehydrogenase (488 IU/L). A 7.4 x 6 x 5.2 cm right ovarian mass that was suspected to be a malignancy was detected on ultrasound (USG) abdomen and pelvis. A computed tomography (CT) scan was performed and revealed a right adnexal mass measuring 7.9 x 6.5 x 5.4 cm that was multiloculated, cystic, and displaced the uterus laterally. Ovarian neoplasm was suggested as a possible diagnosis. Ziehl-Neelsen stain for acid fast bacillus and periodic acid schiff stain for fungal organisms were negative. The patient underwent a total abdominal hysterectomy.

**Figure 1:**
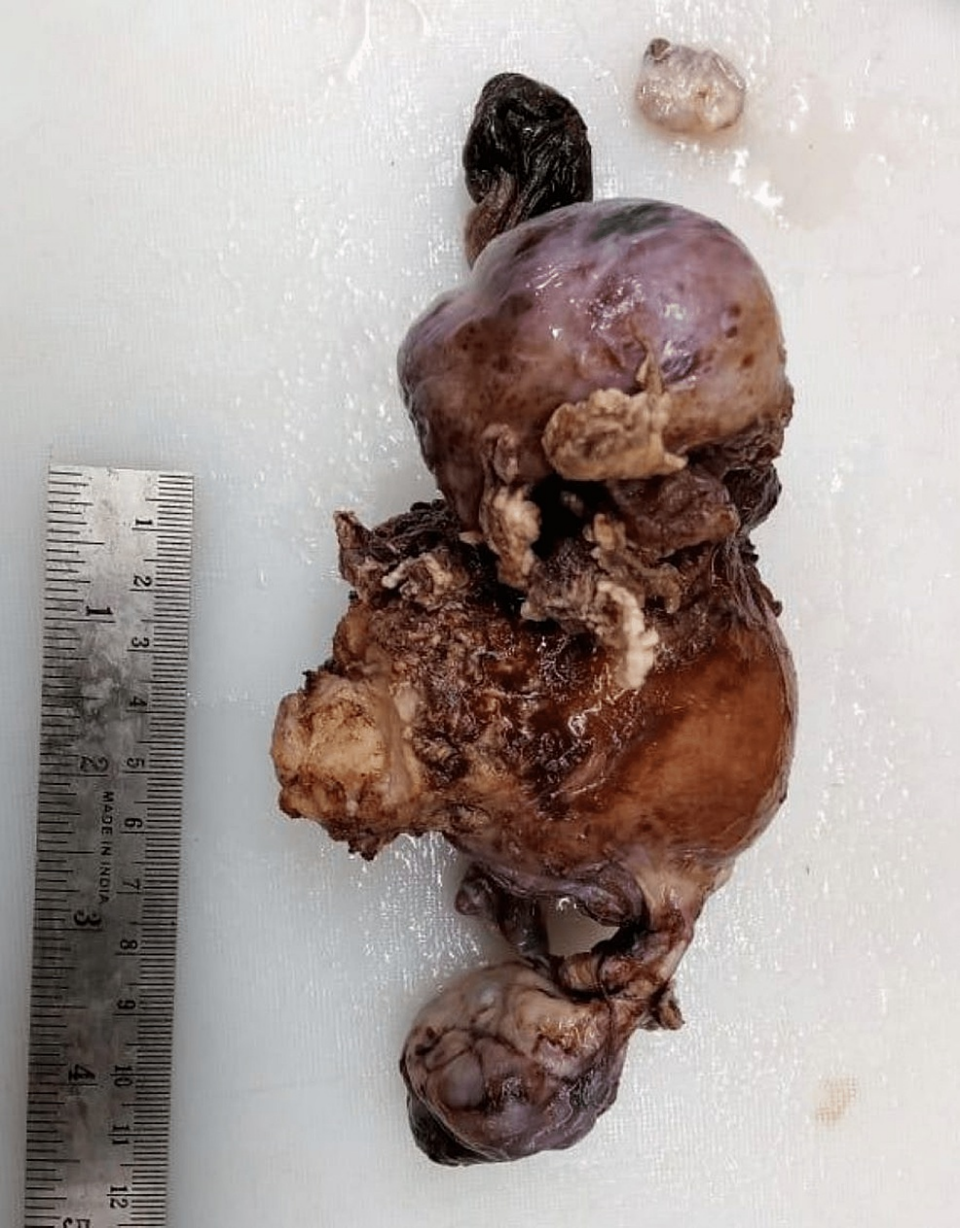
Uterus with cervix with attached bilateral adnexa

**Figure 2:**
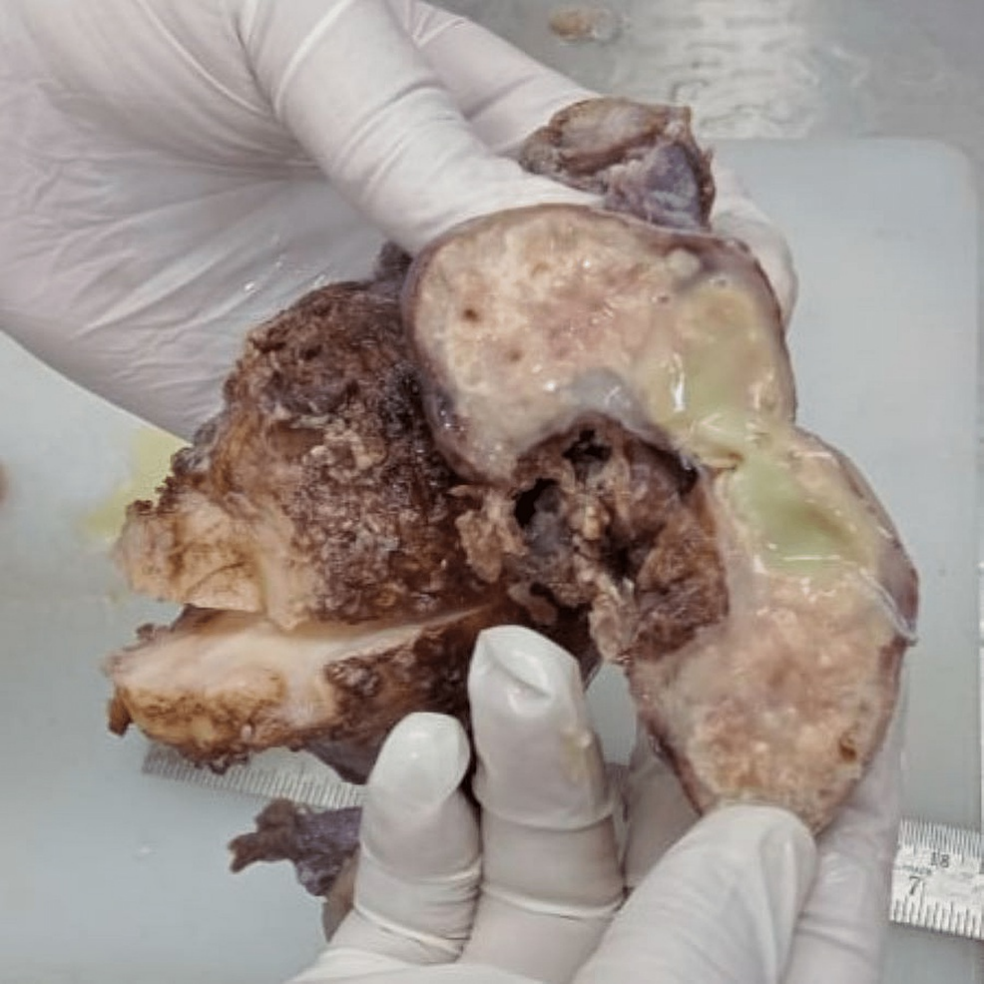
Cut section of right ovary

Grossly, the specimen consisted of uterus with attached bilateral adnexa (Figure 1). The uterus measured 8.5 x 6 x 4 cm. The right ovary measured 5.5 x 4.8 x 2.5 cm, with a greyish-white outer surface. On cut section, right ovary shows greyish-yellow with partly solid and partly cystic areas. From cystic areas, thick yellowish fluid oozed out (Figure 2). The left ovary measured 3 x 2 x 1 cm, and gross examination did not detect any pathology. Uterus and cervix were unremarkable. The right fallopian tube measured 2.5 cm in length and the left fallopian tube was 2 cm long.

**Figure 3:**
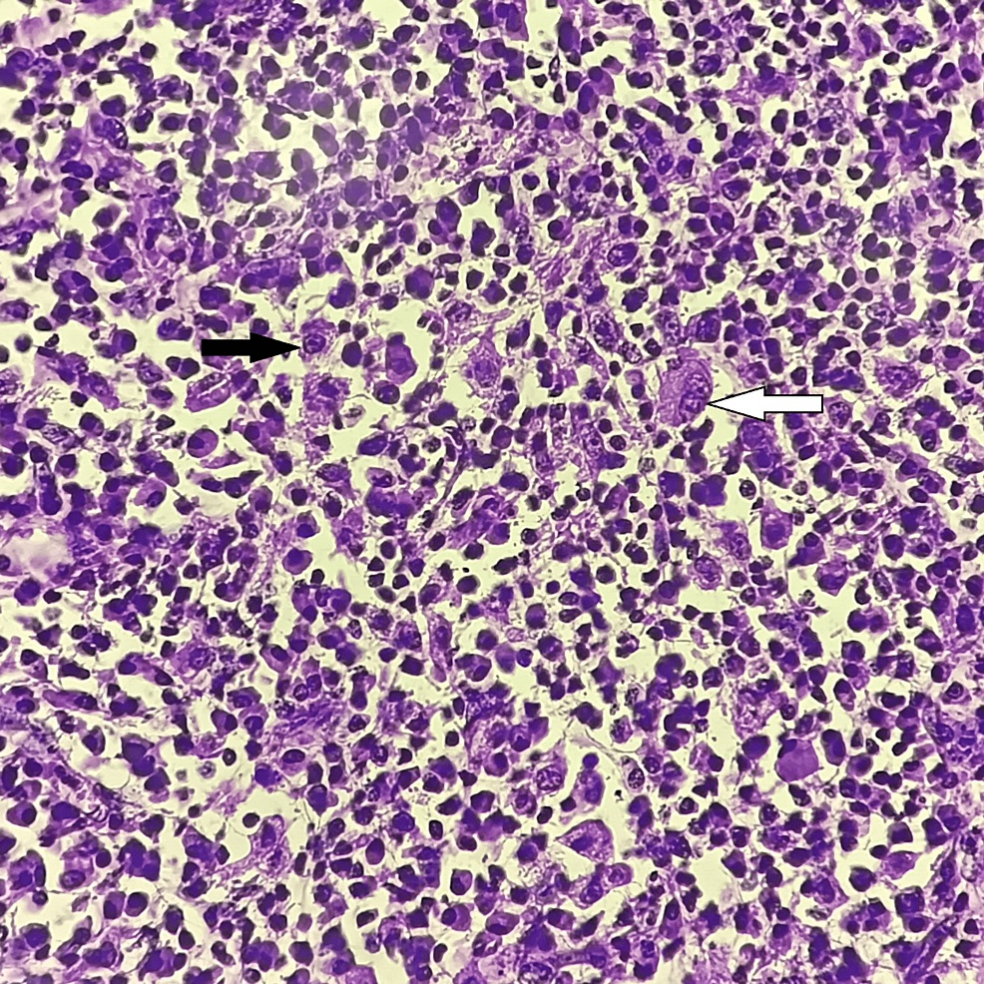
Histopathological photomicrograph of xanthogranulomatous inflammation showing replacement of ovarian stroma by foamy macrophages and histiocytes (H and E, 10X) White arrow showing foamy macrophage and black arrow showing histiocyte

**Figure 4:**
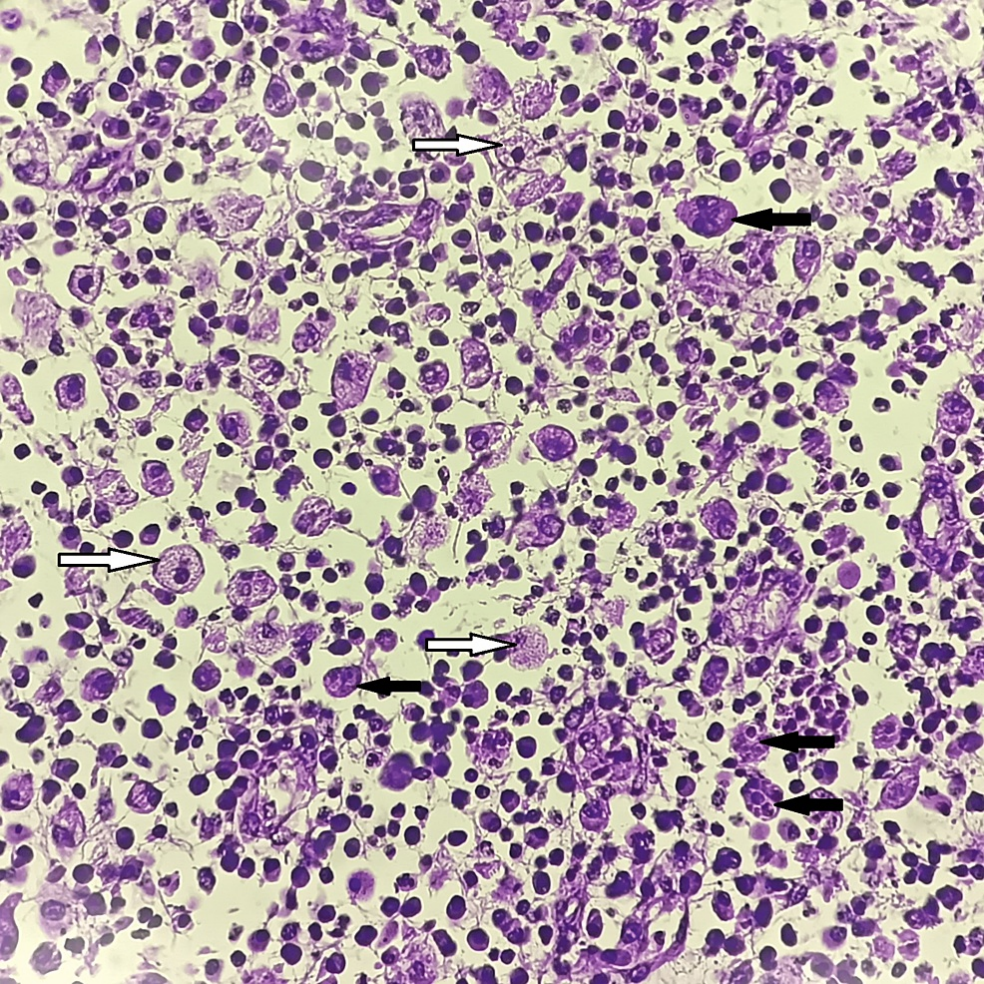
Histopathological photomicrograph of xanthogranulomatous inflammation showing macrophages and histiocytes (H and E, 40X) White arrows showing foamy macrophages and black arrows showing histiocytes

Microscopic examination of the sections stained with hematoxylin and eosin (H and E) was done. Sections studied from right ovary revealed infiltration and replacement of the ovarian stroma by plasma cells, histiocytes, lymphocytes, foamy macrophages and occasional neutrophils. Areas of dense fibrosis are seen along with congested blood vessels (Figure 3, 4). There were no tumour cells or epithelioid cell granulomas. Sections studied from ovarian mass showed no evidence of malignancy. The final diagnosis of xanthogranulomatous oophoritis was made.

## Discussion

Xanthogranulomatous inflammation involving the female genital tract is an uncommon and distinct kind of persistent inflammation with tissue destruction in the affected organs [5]. The etiology and pathogenesis of this condition have been the subject of numerous hypotheses, some of which include endometriosis, infections, intrauterine devices, inborn abnormalities of lipid metabolism in macrophages, drugs (antibiotics), as well as potential combinations of these factors [6]. After the endometrium, the majority of the female reproductive system, including the vagina, cervix, fallopian tubes, and ovary, is commonly impacted by xanthogranulomatous inflammation. *Salmonella typhi, Escherichia coli, Bacteroides fragilis, *and* Staphylococcus aureus* have all been proposed as possible inciting etiological factors for this form of inflammation. Another explanation for this phenomenon is tissue necrosis, which is brought on by a prolonged infection and results in the ongoing release of lipids and cholesterol from the dead cells. The xanthomatous process begins as a result of macrophages phagocytosing these biological components [7].

Xanthogranulomatous oophoritis affects women of childbearing age, with an average age of 35.2 years. A two-year-old girl was the youngest patient with a known case report of xanthogranulomatous ovarian inflammation. It was also reported that a young girl, only 18 years old, had xanthogranulomatous ovarian inflammation [8]. Anorexia, fever, and lower abdomen pain are common symptoms that patients experience when they first come down with pelvic inflammatory disease. A long history of using antibiotics is another factor. It has been shown that xanthogranulomatous oophoritis patients typically have low parity or infertility [9]. Punia et al. claim that untreated pelvic inflammatory disease caused by *Staphylococcus* species have late effects such as xanthogranulomatous oophoritis and salpingitis [10]. In their case report, Shukla et al. also hypothesised a connection between this condition and endometriosis and primary infertility [1]. The significance is that this kind of inflammation might seem like a lump or tumour and can even resemble cancer [11].

According to Walther et al., the presence of foam cells makes malakoplakia an important differential diagnosis. This condition is then separated from xanthogranulomatous oophoritis by the presence of basophilic Michaelis-Gutmann bodies, which are missing in the latter. Michaelis-Gutmann bodies are cytoplasmic concentric calcific structures that are indicative of malakoplakia and are absent in xanthogranulomatous inflammation. Immunohistochemical stains, such as CD68 for histiocytes, CD20 for B cells, and CD3 for T lymphocytes, are useful in making the diagnosis [12]. The inflammatory conditions that come under differential diagnosis include tuberculosis and fungal infections, which are ruled out by special stains and culture studies [13]. Ziehl-Neelsen stain for acid fast bacillus and periodic acid schiff stain were negative in the present study, excluding these possibilities. Tubo-ovarian abscesses are frequently mistaken for ovarian malignancies due to their unique features on magnetic resonance imaging and computed tomography scan [14]. In our situation, ovarian cancer and endometriosis were the differential diagnoses.

## Conclusions

Xanthogranulomatous oophoritis is an interesting case, which mimics ovarian tumours in terms of its clinical and radiological features. The key to effective management is nailing an accurate diagnosis. For its diagnosis, a histopathological examination is crucial. To prevent a misdiagnosis or overdiagnosis of cancer, it is essential to be aware of this entity. In order to prevent radical surgery and for early diagnosis of xanthogranulomatous oophoritis, the majority of patients taking intrauterine contraceptives, those with pelvic inflammatory illnesses, and those with endometriosis require close follow-up.
